# A Regulatory Structure for Working with Genetically Modified Mosquitoes: Lessons from Mexico

**DOI:** 10.1371/journal.pntd.0002623

**Published:** 2014-03-13

**Authors:** Janine M. Ramsey, J. Guillermo Bond, Maria Elena Macotela, Luca Facchinelli, Laura Valerio, David M. Brown, Thomas W. Scott, Anthony A. James

**Affiliations:** 1 Centro Regional de Investigación en Salud Pública, Instituto Nacional de Salud Pública, Tapachula, Chiapas, México; 2 Department of Entomology, University of California, Davis, California, United States of America; 3 Department of Experimental Medicine, Functional Genomics Center, University of Perugia, Perugia, Italy; 4 Pasteur Institute–Cenci Bolognetti Foundation, University of Rome Sapienza, Rome, Italy; 5 Department of Microbiology and Molecular Genetics, University of California, Irvine, California, United States of America; 6 Department of Molecular Biology and Biochemistry, University of California, Irvine, California, United States of America; National Institute of Allergy and Infectious Diseases, United States of America

## Introduction

Sustainable and effective control of dengue is hampered due to a number of factors, including the lack of evidence-based, locally relevant interventions; insufficient information regarding key components of virus transmission and vector ecology; failure to implement precise and efficient surveillance systems; inefficient healthcare systems; ineffective health promotion and outreach resulting in lack of community dialogue and participation; and a paucity of efficient diagnostic strategies and clinical attention [1]. Increased research efforts in response to the complexity of this problem have focused on the development of novel technologies that would enhance existing tools for vector-borne disease prevention [2]–[4]. Genetic strategies to reduce or replace mosquito populations and thereby interrupt transmission of dengue viruses are among the new approaches being considered [5]–[7]. Many of these approaches take advantage of molecular genetic tools to engineer traits that cause lethal phenotypes or confer resistance to the pathogen in the mosquito.

Genetic strategies are being advanced through a series of overlapping domains that inform the decision making on feasibility, safety, efficacy, and acceptability. Although the need to focus on science-based regulation using a risk-assessment framework is gaining support [8], there has been a relative lack of attention on broader community regulations that are explicitly or indirectly required to bring a genetics-based product to the field [3], [9]–[11]. An evidence-based approach would facilitate the integration, efficacy, and acceptability of policy for an intervention strategy.

We addressed the regulatory challenges associated with testing a strain of *Aedes aegypti* engineered to result in population suppression in contained field trials in southwestern Mexico [12]. This large research effort (designated hereafter as the “Project”) combined elements of scientific and social discovery and development as the basis for moving a new technology from the laboratory to the field. Unlike the rollout of other public-health products such as drugs, vaccines, and insecticides, no pipelines exist to move candidate genetically modified mosquitoes (GMMs) from the laboratory through safety and efficacy trials to field deployment. This lack of a preexisting structure made it necessary for the scientists in the Project to play critical, unbiased roles in formulating the product development pathway. The challenge offered a unique opportunity for potential end users and beneficiaries of the technology to be involved from the beginning in product discovery and development. This approach ensures that requirements for safety and efficacy are included as design features engineered into the modified mosquito strains [13]. It is incumbent on the researchers to identify gaps and assist in development of regulatory norms that should be applied to the products they create. These norms include not only statutory regulations but also a broader regulatory environment that addresses the needs and concerns of all communities in which the product will be applied. We describe here the regulatory and social structures used for obtaining approvals in Mexico. This review of our approach is intended to stimulate analysis and dialogue that will help refine regulatory practices of genetic-based strategies for vector-borne disease control.

## Regulatory Domains for the Discovery and Development of Genetically Modified Mosquitoes

An initial challenge of the Project was identifying relevant communities [14]. No consensus existed as to what comprises a relevant community to engage for a GMMs research project, nor were there any widely accepted methods for identifying their members. We adopted a definition (modified from [15]) in which the community consists of all those individuals who share the identified risks and/or will benefit from the outcome of the proposed research project. In this context, the community coalesces as a result of the project and evolves continuously as it progresses through conceptualization, discussion, and implementation [16]. The community is formed ultimately by those individuals, groups, organizations, and agencies that have legitimate interest in the research, and therefore they must be engaged in an effective and timely manner.

**Figure 1:**
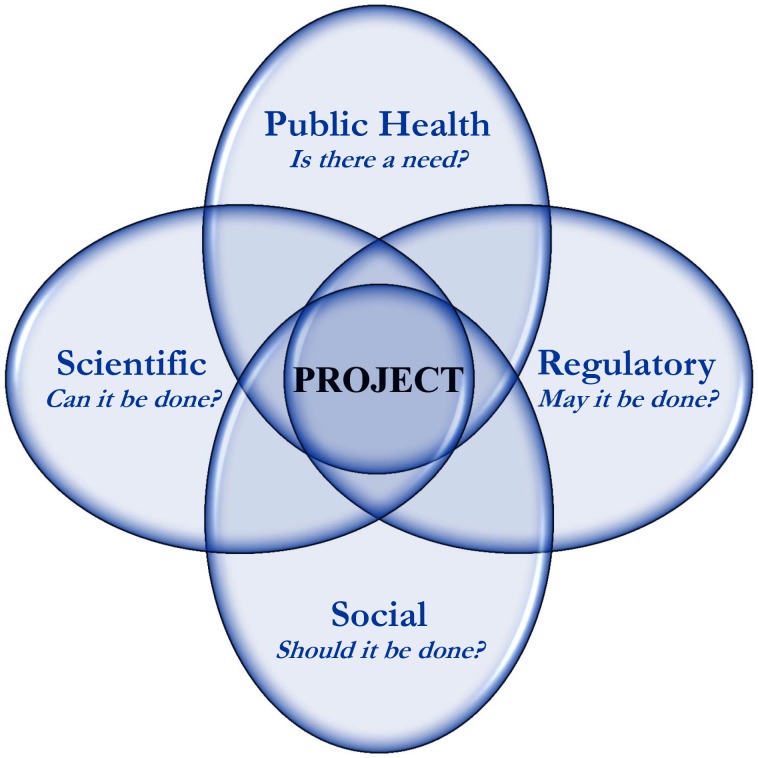
Schematic representation of activity domains for the discovery and development of novel tools for public health. Each circle represents an activity domain in which specific questions must be answered before a novel public health technology is adapted for use. The first domain, public health, generates the question of need. The scientific domain seeks to develop the novel products. The regulatory domain comprises those elements that are needed for statutory permission to deploy a novel product. Finally, the social domain must agree that the new product should be implemented. A successful project involves the finding the positive overlap of all of these domains.

Successfully moving a novel technology from the laboratory to practical application depends on meeting the specific demands at the intersection of a number of activity domains (Figure 1). In addition to validating the relevance and merit of the technology, these domains also guide the organization of the operational components of product development. The first domain, public health, provides the evidence base for justifying the need to intervene for a defined public health risk. The challenge of sustaining dengue disease prevention using current strategies and approaches clearly identifies a need for new strategies for implementing existing tools as well as the development of novel tools. The constituents in this domain include persons at risk for the disease, members of primary healthcare systems, scientists, non-governmental organizations, private businesses, and international agencies involved in the detection and analysis of disease.

Researchers in the scientific domain work to meet the public health need by developing novel strategies and products. The task for our Project was to determine whether it is possible to engineer mosquitoes that carry conditionally lethal genes that would result in the suppression of target vector populations. This involved laboratory development of such strains, analyses of the impact of transgenes on the ecology and population dynamics of natural and engineered populations, and eventually, documenting their safety and efficacy. Results achieved in the laboratory prior to our contained field trial were encouraging [6], [17]. Investigators demonstrated proof of principal for a number of genetically modified lines with specific phenotypes, and efforts were initiated to determine whether laboratory successes could be reproduced in more natural field conditions [12], [18]–[20]. This domain provides part of the evidence base for risk assessment and the technical requirements of the regulatory processes.

The regulatory domain addresses technical statutory and contractual obligations and requires the existence of a functional system that has the knowledge, capacity, and authority to review, monitor, and grant permission for research on genetically modified organisms (GMOs) [21]. Laboratory and cage trials, followed by open-field releases, depend on the presence of one or more sanctioned oversight bodies capable of granting the necessary approvals and the ability and capacity of the researchers to meet and maintain compliance with regulatory requirements.

The social domain is less systematized than institutional technical regulatory processes because it is highly dependent on cultural factors such as traditions, practices, and cohesion, as well as other community interactions and structures at local levels. Local communities that may be exposed during field trials (open- or contained-field trials) need to be made aware of research and risk considerations and be informed on the overall and specific context of the research. Local populations are engaged implicitly as a result of the presence of the research team among them. The social domain is a population-based regulatory system guided by cultural, political, economic, gender, equality-related, and pragmatic components. This domain is inherently part of the statutory and technical regulations since social concern is one of the components that drive the establishment of technical regulatory systems. In order to map, engage, and address social concerns, information exchange and dialogue with the community at multiple levels and scenarios are needed, and they ensure development of respectful interactions, participation, and eventually, trust between scientists and end users [22]–[25]. These activities build confidence in the community for the Project and research team through respectful interchange, engagement, transparency, dialogue, and insertion into community life and practices [16], [26]. The result is to achieve a pragmatic “informed consent” through participation at all levels, for continued development, adaptation, and ultimately, sustainable implementation of the new technology. Proposed novel approaches and products may be adapted as a broader representation of communities is engaged and new concerns are expressed.

Community engagement activities by Project members were developed in conjunction with multilevel ethnographic information and allowed Project scientists to establish an open dialogue and information exchange at individual, family, and collective levels [14], [16]. This established the basis for explicitly requesting community approval and input for creating guidelines for Project activities, separate from federal and institutional regulatory approvals. Moreover, risk assessment and cost/benefit analyses use input from the social domain to address whether genetic approaches have the capacity to fulfill an unmet need for an affordable, safe, acceptable, and efficacious tool that has public-health benefit. The domain generates analyses of community attitudes and representations that incorporate cultural and equality issues, key components of community regulatory processes. It also integrates both technical and community-based regulatory processes as outreach that encourages a broad range of people in the community to join the dialogue. Community awareness and participation support proper adherence to technical regulations and the development of regulatory norms by raising additional social or cultural issues, and they provide opportunities for constituents to express opinions on how human and environmental vulnerability can be minimized [11].

Criteria for field site selection procedures are derived from all of the domains. Criteria for our Project included the identification of candidate countries that had the necessary regulatory structures to oversee GMM research, research institutions with appropriate experience in vector-borne disease research, and research regulatory structures (for example, Institutional Review Boards [IRB], ethical review boards, Institutional Biosafety Commissions [IBCs], and animal care and use commissions [14]). Existing community structures and collective discussion and decision-making were considered beneficial for complying with informal regulatory processes overlapping the social domain. Mexico was deemed after extensive review to have the many basic technical and community regulatory features needed for the Project.

## Technical Regulatory Framework

The features of the technical regulatory processes documented herein are a public record of the highest research and ethical standards of the Project to ensure public health and environmental safety. These features include compliance with regulations originating in national legislation and enforced by established agencies, procedures, and community structures, as well as those motivated by and implemented specifically for this Project. We expect that the fundamental elements of these technical regulatory structures can be adopted by countries considering the use of GMMs for disease control, although we anticipate that they will require validation or adjustments for local sociocultural environments. Respectful insertion into local social norms and structures is a key component for an integrated approach to the field trials.

A number of publications document Project background, laboratory achievements, and expectations for the technical feasibility of testing the performance characteristics of a flightless-female strain of *A. aegypti*, OX3604C [6], [14], [17]. These publications chronicle the development of the rationale and performance characteristics of the prototype strain and provide the initial data for risk assessment analysis. The contained-field trial in Mexico [27] required that the Project design and build large field cages in which the experiments were to be conducted. This necessitated that a site be available for large cages; located outside a major urban area or grouping of houses with historical evidence of dengue transmission and presence of *A. aegypti*; and where, as a component of risk assessment, containment of experimental populations and monitoring for potential escaped organisms was sensitive, effective, and acceptable. The nature of the field trial brought the research into a local community in a coastal, rural village, Rio Florida, in Tapachula County, Chiapas, 46 km from the Guatemalan border. It took almost three years to meet all of the regulatory, social, and infrastructure requirements needed to initiate the cage trials.

**Figure 2:**
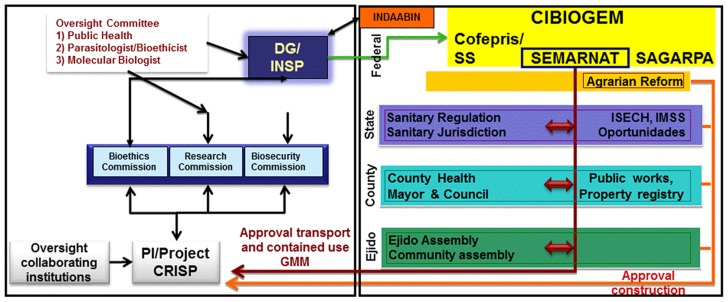
Schematic representation of the regulatory pathway for research involving genetically-modified mosquitoes. Activities of the Project principal investigator and collaborating institutions (oversight collaborating institutions) are coordinated in Mexico by the principal investigator at the Centro Regional de Investigación en Salud Pública (PI/Project Crisp). CRISP also is the regional center of the researchers conducting the work at the field site. The director general of the Instituto Nacional de Salud Publica/National Institute of Public Health (DG/INSP) is the titular head of the collaborating investigators in Mexico. The oversight committee reviews ethical, biosafety, and scientific practices used in the project and makes recommendations to the DG/INSP regarding procedures or adherence of the project participants or internal commissions to appropriate standards. The Bioethics, Research and Biosecurity Commissions are in-house INSP regulatory bodies. CIBIOGEM (Comisión Intersectorial de Bioseguridad de los Organismos Geneticamente Modificados [Intersector Commission for Biosafety of Genetically Modified Organisms]) is the federal-level commission that formulates and coordinates policies in matters related to the biosafety of genetically modified organisms. CIBIOGEM comprises the heads of SEMARNAT (Secretaria del Medio Ambiente y Recursos Naturales [Environmental and Natural Resources Secretary]), SAGARPA (Secretaria de Agricultura, Ganaderia, Desarrollo Rural, Pesca y Alimentación [Agriculture, Livestock, Rural Development, Fisheries and Food Security Secretary]), Cofepris (Comisión Federal para la Protección contra Riesgos Sanitarios [Federal Comision for the Protection against Sanitary risk])/SS (Secretaría de Salud [Secretary of Health]), and a number of others listed in the text. SEMARNAT is the authority to which the Notification (*Aviso*) was submitted regarding the importation and contained research on genetically modified organisms and that which granted a permit for the collection of native fauna to monitor transgenes. The red arrows show the permit ratification levels and information flow. State-level Sanitary Regulation (IMSS, Instituto Mexicano de Seguro Social [Mexican Social Security Institute]; ISECH, Instituto de Salud del Estado de Chiapas [Chiapas State Health Institute]) monitors and enforces health regulations at this level and ratifies SEMARNAT's permit for genetically modified organisms. County-level Health Committee ratifies ISECH/Cofepris approval for use of genetically modified organisms. Agrarian Reform grants changes in land classification and permission to purchase land and this informs the regional authorities (orange arrow and lines). The Ejido Assembly gives local permission to sell land to INSP and INDAABIN (Instituto de Administración y Avalúos de Bienes Nacionales [Appraisal and Administration of National Property Institute]) grants permission for property purchase by INSP. Catastro (land registry) gives land titles and obras publicas (public works) grants construction permits.

The technical regulatory pathway for the Project contained two principle components. The first linked a multilevel system of agencies regulating and monitoring GMOs and environmental risks by means of Mexican institutions (federal, state, county, and other) (Figure 2). The second component comprised academic regulatory committees within all collaborating institutions, whether Mexican or from other countries (Instituto Nacional de Salud Publica [INSP] and its regional center, Centro Regional de Investigación en Salud Pública [CRISP]; University of California, Davis; University of California, Irvine; Colorado State University; North Carolina State University; Cornell University; and Oxitec Ltd). At least three separate lines of communication linked the two components. The Project and collaborating institutions were placed administratively in the academic component overseen by INSP (labeled PI/Project CRISP in Figure 2). Project activities were reviewed by Institutional/Internal Review Boards (IRBs), Institutional Biosafety Committees/Commissions (IBCs), and animal care and use committees at collaborating institutions and by the INSP Regulatory Commissions that oversee ethics, research, and biosecurity. A coordinating council of the three INSP Commissions was created to review jointly and approve progression through the Project stages and expedite review of specific amended experimental protocols. The president of the Research Commission was the lead for all three INSP components, and communications between these and the Project were routed through this office. In addition, a Project-dedicated External Oversight Committee was charged by the director general (DG) of INSP to review and report all ethical, biosafety, and scientific activities and to make recommendations on procedures and adherence of the Project participants and INSP commissions to appropriate standards.

## Federal Regulatory Structures Relevant to Public Health Research Using Genetically Modified Organisms

Testing of a GMM strain in Mexico required meeting the provisions of previously established laws and regulations (Table 1). Risk assessment and potential impact of GMOs other than those that fall under the Secretary of Agriculture, Livestock, Rural Development, Fisheries, and Food (Secretaría de Agricultura, Ganadería, Desarrollo Rural, Pesca, y Alimentación, SAGARPA), which include GMOs for agricultural use, forestry, aquaculture, and plant and animal health, is regulated by the Secretary of the Environment and Natural Resources (Secretaria del Medio Ambiente y Recursos Naturales, SEMARNAT). SEMARNAT is one of six members of the Inter-secretarial Commission on the Biosafety of Genetically Modified Organisms (Comisión Intersecretarial de Bioseguridad de los Organismos Genéticamente Modificados, CIBIOGEM, www.cibiogem.gob.mx). The Directorate for Environmental Risk and Impact (Dirección General de Impacto y Riesgo Ambiental, DGIRA) within SEMARNAT is the office responsible for registry of the Notification (*Aviso* in Spanish) for contained maintenance, use, or testing of GMOs, as well as for issuing permits for environmental releases of GMOs within their mandate. Communications with SEMARNAT and DGIRA officials were initiated two years prior to the Project's request for approval of the contained-field trial to discuss the role of genetic strategies in public health, the proposed trial of a transgenic *A. aegypti* strain, and the nature of the GMMs to be tested.

**Table 1:** Mexican federal regulatory agencies and their laws and regulations relevant to the contained-field trial of genetically modified *A. aegypti*.

Federal Secretariat	Law or Guideline	Website/document record
**Public Education (SEP)**	Law for Science and Technology	www.diputados.gob.mx/LeyesBiblio/pdf/242.pdf
**Health (SS)**	General Health Law	www.diputados.gob.mx/LeyesBiblio/pdf/142.pdf
	Regulations for the General Health Law in matters of research for health	www.salud.gob.mx/unidades/cdi/nom/compi/rlgsmis.html
	INSP Guidelines for the Research, Ethics, and Biosecurity Commissions	www.insp.mx/normateca
**SEMARNAT**	Notification (*Aviso*) procedure for the Biosafety Commission and contained use of GMOs	http://www.cofemer.gob.mx/wwwroot/BuscadorRFTS/DatosGenerales.asp?homoclave=SEMARNAT-04-14&modalidad=0&identificador=1417218&SIGLAS=SEMARNAT
**CIBIOGEM**	Law on Biosafety of Genetically Modified Organisms in Research Centers	http://www.cibiogem.gob.mx/Norm_leyes/Paginas/default.aspx
	Regulations for the Law on Biosecurity of Genetically Modified Organisms	http://www.cibiogem.gob.mx/Norm_leyes/Paginas/default.aspx
**SAGARPA**	Federal Law for Animal Health	www.diputados.gob.mx/LeyesBiblio/pdf/LFSA.pdf
	Request for transportation of fauna and genetically modified organisms	http://www.sagarpa.gob.mx/tramitesyServicios/Paginas/senasica02.aspx?SelectedList=Direcci%c3%b3n%20General%20de%20Salud%20Animal
**Gobernación**	Agrarian Reform Law	http://www.diputados.gob.mx/LeyesBiblio/ref/lagra.htm
**United States Office of Laboratory Animal Welfare assurance (OLAW)**	Standards for humane care and use of laboratory animals	Welfare Assurance number A5821-01

CIBIOGEM is the federal-level commission that formulates and coordinates policies in matters related to the biosafety of genetically modified organisms. CIBIOGEM comprises the heads of SEMARNAT and the Secretariats SAGARPA, Health (Secretaría de Salud, SS), Public Education (Secretaría de Educación Pública, SEP), Finance and Public Credit (Secretaría de Hacienda y Crédito Público, SHCP), Economy (Secretaría de Economía, SE), and the director general of the National Council for Science and Technology (Consejo Nacional de Ciencia y Tecnología, CONACyT). The Law on Biosafety of Genetically Modified Organisms (http://www.cibiogem.gob.mx/Norm_leyes/Paginas/default.aspx; English translations are available at http://www.cibiogem.gob.mx/eng/Regulatory-Framework/Paginas/default.aspx) regulates the activities of contained and confined use, experimental release, release in a pilot program, commercial release, trading, importation, and exportation of GMOs. The objective of the law is to prevent, avoid, or reduce the potential risks that these activities might pose to human health, the environment, and biological diversity, or to the health of animals, plants, and aquatic organisms. This law also provides specific mandates promoting scientific research on biotechnology, biosafety, and the use of GMOs to solve social problems and assist national agricultural policies. The Regulations for Biosafety of Genetically Modified Organisms provide the details for the proper application of the Law.

Although a permit *per se* is not required for contained field research on GMOs, the Notification (*Aviso*) of all research projects is required to be registered either by DGIRA/SEMARNAT or SAGARPA, depending on the GMO and its use. GMOs produced, stored, and tested for teaching purposes or for scientific and technological research are subject to the notification procedure, and must be handled under approval and monitoring of an IBC in accordance with current regulations. Notification (*Aviso*) requires submission and complete disclosure of (a) the characteristics of the organism to be tested, (b) the risk level assigned to the organisms, (c) all transport procedures within and to the country, (d) the location, design and features of facilities where the GMOs will be handled or assayed, (e) the existence of a Biosafety Commission and an internal subcommittee within that commission with at least three experts in molecular biology, (f) all efficacy testing procedures, (g) standard operating procedures (SOPs) for confinement and personnel protection procedures (not mandated for initial procedures but requested during technical evaluations), and (h) maintenance of trial logs and activities, as well as assay records for review when requested (Mexican law mandates maintaining research records for seven years). Review and approval of the Notification (*Aviso*) for the contained trial of OX3604C included opinions by the two technical advisory agencies of SEMARNAT, the National Institute for Ecology (Instituto Nacional de Ecología y Cambio Climático, INECC) and the National Biodiversity Council (Comisión Nacional para el Conocimiento y Uso de la Biodiversidad, CONABIO) (Table 2). The process took three months following protocol submission and was the first approval in Mexico for contained field trials of a GMO for public health.

**Table 2:** Regulatory processes for approval of the contained-field testing of genetically modified *A. aegypti* in Rio Florido, Tapachula, Chiapas, Mexico.

Activity	Federal regulatory	Academic regulatory	Community regulatory
Notification (*Aviso*) of the modified INSP Internal Biosafety Commission	S.G.P.A/DGIRA/DG/3917/08 (11/21/08)	Internal Biosafety Commission INSP registered with CIBIOGEM and includes molecular biology subcommittee (5/1/08)	INSP Research Boards have oversight by the INSP governing board, with members from a broad range of society
Research project review by internal commissions of INSP (research, ethics, biosafety)	-	Approval by each commission, review based on five project stages or specific components, with yearly IRB review/renewal. (6/5/08)	Copy of documents submitted to state, regional, local, environment, health, and civil authorities and presented in outreach meetings (local and national)
Institutional review of research protocols that involve human subjects	National Institute of Health (US)	Protocols reviewed by Institutional Review Boards (IRB) at Cornell University, University of California Davis and University of California Irvine	-
Institutional review of research protocols that involve pathogens and/or recombinant DNA	National Institute of Health (US)	Protocols reviewed by Institutional Biosafety Committee (IBC) at Colorado State University	-
Institutional review of research protocols that involve vertebrate animal use and care	National Institute of Health (US)	Protocols reviewed by Institutional Animal Care and Use Committees (IACUC) at Colorado State University, Cornell University, and University of California Davis	-
Notification (*Aviso*) to DGIRA/SEMARNAT for the “First use in research laboratory and field installations for handling the genetically modified *A. aegypti* strain RIDL OX3604C for the purpose of controlling wild populations”	Submitted to DGIRA 8/17/09; technical review by INE (Instituto Nacional de Ecología [National Institute of Ecology]) and CONABIO; approval (11/6/09) for maintenance of transgenic strain in insectary, and caged-field trial of OX3604C (S.G.P.A./DGIRA/7074/09; S.G.P.A./DGIRA/DG/8105/09). Technical inspection by INE (9/3/11). Final report of study to DGIRA and CIBIOGEM (3/30/12)	-	The content and experimental procedures contained in Project were explained in many outreach meetings, and all copies of federal approval documents submitted to state, county, local, environment, health, and local community authorities.
Importation permit for dry eggs of *A. aegypti* from England (OX3604C) and US (GDLS1 and 2)	Submitted to SAGARPA 12/14/09. Approved 1/26/10	-	Copy of documents submitted to state and local environment and health authorities, and local community authorities.
Procedures Manual for Maintenance and Testing of Genetically Modified *A. aegypti*.	First and final versions submitted to DGIRA and CIBIOGEM: used for technical evaluation of project by INE (Sept. 2011); final version 4/7/10, additions or modifications finalized (3/30/12, not mandatory)	Not requested; submitted and modifications reported in each Biosecurity stage report. Published in INSP website norm and manuals directory	Copies of these procedures documents were given to all engaged communities for comment, to motivate discussion and resolution of doubts and for “informed” approval to proceed
Public domain under Secretariat of Agrarian Reform to privatize land for purchase by INSP	Initiation of activities in Ejido Rio Florido (3/31/07), approval for public domain (9/11/08)	-	Integrated outreach for purchase of land and dialogue regarding dengue, its control, genetic strategies, and the Project
Permit for a government institution to purchase land	INDAABIN approved (26/06/08)	-	-
County property title INSP	Catastro (6/29/09)	-	-
Environmental impact evaluation for county construction permits	Received (07/03/08)	-	-
County construction permit for field site facilities	Facilities constructed (12/15/08-9/6/09), cage containment certified (4/29/10)	-	-

Regulatory compliance during the Project involved maintenance of records and data on trial experiments (conducted by a biosafety procedures monitoring control manager), as well as strict adherence to a containment monitoring program. Depending on the length of trials, INECC conducts at least one inspection of the experimental site and all mosquito monitoring records. This inspection of the Project's field cages and corresponding review of all records during the trial returned no comments, warnings, or further requirements for information. Upon completion of the trial, a full report with copies of monitoring registries, trial outcome, and complete disclosure of the escape monitoring program and results was submitted to DGIRA and CIBIOGEM. In addition, the project disclosed the final disposition of remaining GMMs, whether maintained in an active or inactive (frozen) state.

The transport of any product of animal-origin, infectious agent or vector is regulated by SAGARPA, specifically, the Directorate for Import, Export, and Livestock Services (Dirección de Importación, Exportación, y Servicios Pecuarios, http://www.senasica.gob.mx). A notification of importation of a GMO is required if it is not developed in Mexico (and for transport within if developed in Mexico), and project-specific importation regulatory requirements from SAGARPA and Customs (in the Secretary of Finance and Internal Revenue, SHCP) can be obtained only after the research group has complied with the Notification (*Aviso*) procedure and has been registered by the appropriate authority.

Other federal laws and regulations relevant to the contained trial were related to establishing test-site facilities, and thus assuring the legal acquisition of a suitable piece of land by INSP (being a federal institution). The Agrarian Reform Law (Ley Agraria; http://www.diputados.gob.mx/LeyesBiblio/ref/lagra.htm) addresses issues related to community land use, regulation of collective land ownership, and community decision making (*ejido*). Hence, the testing or use of GMMs in field cages was linked implicitly to insertion into agrarian reform and community norms. This engendered an additional need for outreach information on dengue, dengue control, genetic strategies, and research on a laboratory-engineered vector of dengue.

Negotiating the field-site acquisition involved interactions with both legal and community regulatory structures, due to the collective nature of *ejido* land and the need to adhere to processes mandated by the *Asamblea Ejidal* (required by the Agrarian Reform Law). Agrarian laws permit the sale of collectively held *ejido* land only if ≥51% of titled members (*ejidatarios*) approve. Approval often is not possible due to the members' conviction of the importance of maintaining the *ejido* sociopolitical structures for collective land holding and decision making. Town meetings are held, during which there is a thorough review of the potential “new neighbors” and their objectives for purchasing the land (in our case for the Project and beyond), followed by lengthy discussion among only the *ejidatarios*. If there is a positive vote to remand the designated land to public domain, the Secretariat of Agrarian Reform (Secretaría de la Reforma Agraria, SRA) registers, and under the law, approves the sale. Three assembly meetings were held to exchange information and dialogue on issues related to dengue, vectors, control, modified organisms, risk assessment, technical and community regulatory issues and permits, and research processes, after which there was a majority vote to allow the INSP to purchase a property on the edge of the Rio Florido community. This was the first approval for sale of collectively owned land in this community.

INSP is part of the Mexican federal government (Health Secretariat), so following Agrarian Reform approval to allow the sale of the land, it needed internal government permission from the Instituto de Administración y Avalúos de Bienes Nacionales, Secretaría de la Función Pública (INDAABIN/SFP) to secure the purchase at the proposed price. This document was required under county regulation for land title registry.

## State and Regional Regulatory Structures

Although there is no legislation at the state level in Mexico mandating regulatory issues regarding research, public health, environmental, or issues with GMOs, state authorities are responsible for complying with federal approvals and permits and must be aware of any projects operating within their state territory. Once Notification (*Aviso*) of the proposed contained field trial was made to DGIRA/SEMARNAT and registered with CIBIOGEM, and as part of the multilevel outreach and engagement to assure appropriate institutional interactions by the Project, all copies of federal and institutional documents and approvals were provided to state environment and health secretaries, the Federal Environment Delegate in Chiapas, the State Health Secretariat, and Sanitary Regulation. Project documents also were presented to regional public health and environment coordinators, as well as county authorities (mayor and council members), within the framework of engagement activities. Most of the county institutional authorities requested information and held meetings to inform and query the group regarding the Project. The mayor of Tapachula held a council meeting to debate the proposed project and, in the absence of dissenting opinions, gave verbal approval for the Project to be conducted in the county.

## Academic Regulatory Committees

The law for science and technology (www.diputados.gob.mx/LeyesBiblio/pdf/242.pdf) regulates research and research capacity and management in Mexico. A specific law, the General Law on Health (Ley General de Salud, www.diputados.gob.mx/LeyesBiblio/pdf/142.pdf), mandates the population's right to protection if the research is on public health topics. Guidelines are provided in the Regulation for Health Research (Reglamento de la Ley General de Salud en Materia de Investigacion para la Salud www.salud.gob.mx/unidades/cdi/nom/compi/rlgsmis.html). INSP is one of the few research institutions in Mexico with full research review boards comprising technical, biosafety, and ethics (IRB) commissions. GMO regulations in Mexico specifically require inspection or monitoring of any laboratory-based or contained-field tests of GMOs by an Internal Biosafety Commission that includes at least three molecular biologists and is registered formally through the Notification (*Aviso*) procedure with the corresponding authority. The INSP Biosafety Commission hence created a molecular biology subcommittee and registered it with DGIRA. Federal environmental (SEMARNAT) and animal welfare and transportation (SAGARPA) agencies communicated to and from the Project only via the Biosafety Commission. One exception approved by SEMARNAT was for the regulation regarding who should be advised in case of an inadvertent GMM escape. Since regulations stipulate advising authorities within 24 hours of evidence of an escape, the procedure was modified so that, should an escape occur, the principal investigator (PI) was approved to directly advise DGIRA, with copy being sent to the INSP Biosafety Commission.

All local community engagement programs accompanying the contained field trial required full IRB approval, annual renewal of ethics certification by project leaders (all senior Project scientists complied), and detailed annual review of appropriate consent considerations required by international and national research ethics review. Project stage reports were reviewed by all three INSP Commissions, and approval given together for progression to the next stage in the research program. One technical issue (incomplete homozygosity of the transgene in the parent colony) during the Project required informing and providing comments to the Commissions on the potential impact on the original risk assessment profile. While this event did not involve an inadvertent release of any GMMs, the Commissions recommended temporary suspension of the trial until sufficient evidence could be gathered to demonstrate no significant alterations to the biological profile of the mosquito strain. Following open discussion among all Project and Biosafety Commission members and disclosure of all evidence, there was unanimous approval to continue the trial.

In addition to the federally-required SOPs on confinement and personnel protection procedures, the Project prepared detailed manuals for the transport, breeding, maintenance, and testing of genetically modified *A. aegypti* to supplement information made available to the INSP regulatory commissions. The manuals included all modifications or additions through the termination of the trial and were prepared in both Spanish and English. This manual was approved and incorporated into the Biosafety Commission and institutional-norm registry.

## Discussion

The introduction of novel technologies should be accompanied by appropriate technical and community regulatory requirements. An integrated approach to their development and testing should result in the availability of safe and efficacious products in a manner consistent with the highest technical and ethical standards. Particular challenges are presented by technologies for which there is little background, experience, or previously developed, specific pathways to guide the transition from the laboratory to the field. The development of a GMM line for control of pathogen transmission is among these technologies. Proponents must simultaneously develop the science and contribute meaningfully to community dialogue regarding if, when, where, and how it could and should be applied. Part of this process is to meet the legal, technical, and community regulatory requirements for testing and use. Rigorous site-selection procedures identified Mexico as having the necessary capacity to support the first field-cage tests on GMM vectors of dengue [14]. While we do not expect every country to adopt these same requirements, we anticipate that our experience can assist in the planning for future efforts to test these kinds of technologies.

Given the early involvement and contributions to developing genetically modified plants, Mexico has been a leader in government regulation and oversight of research, testing, and use of genetics-based technology. As a result, our efforts in Mexico were expedited by the presence of three critical regulatory and community features, the combination of which was unique at the time when our Project was initiated. The first was the existence of CIBIOGEM, the federal-level body that oversees the coordinated technical review of the proposed research by all secretariats and agencies expected to have some authority in the process. Specifically, authorities for public health, agriculture, and environment and natural resources are represented in the review process. Thus, questions and concerns of the impact of the research on human and animal health and the environment would have sufficient technical review. The benefit to the Project from such a structure was that CIBIOGEM was a single, defined body with appropriate expertise to which we could communicate regarding the experimental trial, and through one of the constituent agencies, SEMARNAT, receive the necessary registration and review.

The second major factor was the presence of a world-class collaborative scientific institution, INSP, and its on-site regional center, CRISP, which contributed the expertise, scientists, outreach experience, and management that were essential for coordinating testing of this new technology. They also provided the technical and ethical scope of review that was essential for IRBs, IBCs, and animal care and use committees. Special provisions applied by INSP for this Project included specific, coordinated discussions of review procedures and the addition of an oversight committee established by the director general. These structures contributed at all levels to the assurance that issues with genetic engineering and ethical engagement of communities were addressed satisfactorily.

The third critical factor arose out of the Mexican agrarian reform, current modifications of original landholding laws, and the collective decision-making processes of the *asamblea ejidal*. One of the primary impacts of the 1910 Mexican Revolution was to return decision making regarding territorial issues to those who worked and lived on the land. The resulting reform linked all territorial and community decision making to the collectively administered *ejidos*. Recent secondary land reform provides the opportunity for collectively owned *ejidos* to permit land privatization and sale, and this has contributed to a recent shift from total collective ownership to privatization. Because collective community decisions are dependent on the *asamblea ejidal*, they too are currently immersed in an evolutionary process, shifting from traditional, patriarchal decision making by the *ejidatarios* to the more modern, vertical, political party and public administration structures. Both traditional and modern public administration decision making are operating concurrently, especially at a local level. However, the *asamblea ejidal*, because of its historic acceptability, is still considered in many rural communities to be the key community regulatory body. Community approval was necessary for INSP to acquire land for the trial, and part of the deliberative process prior to granting permission was for the INSP and the Project to communicate its intentions for initial as well as potential future use. Thus, the *asamblea ejidal* was an integrating entity for all regulatory components at the local level. Importantly, their permission to sell the land met the criteria of community consent and was taken as an explicit approval for Project activities.

The philosophical approach of the scientific group developing GMMs will influence how a project interfaces with the multiple political and economic forces that drive outreach and public dialogue at a national level and the community regulatory processes at a local level. While there is clear leadership and appropriate processes regarding technical regulatory procedures at the federal level, this is not the case for community regulatory processes. We expect that finding analogous structures for collective decision making in other countries will be difficult, but this highlights the need to tailor testing and development of novel genetic strategies to all levels of each society, their many communities, and according to the particular public health and environmental context.

The organized coordination of civil, sanitary, legal, environmental, community, experimental and administrative components of field testing GMOs will likely have phases of discovery, development, and scale-up. We can expect that processes will include iterations and adjustments. Add to this the global nature of such a project, with financing and stakeholders having multiple national, cultural, scientific, and institutional experiences, norms, and perspectives, and the testing of a novel genetic-based technology can quickly become a complex process. Therefore, a project designed to test the public health potential of genetically modified products requires careful attention at all levels to continuous, open, and proactive communication. Information exchange and dialogue should be transparent. There should be recognition and participation by all community members, which will lead to increased confidence and mutual respect among the community and institutional efficiency, adaptability, and compliance. It is our hope that the experiences of those who embraced these complex regulatory challenges will contribute to increasingly more effective technical and community approval and, ultimately, disease prevention.
